# Lengths of the C-Terminus and Interconnecting Loops Impact Stability of Spider-Derived Gating Modifier Toxins

**DOI:** 10.3390/toxins9080248

**Published:** 2017-08-12

**Authors:** Akello J. Agwa, Yen-Hua Huang, David J. Craik, Sónia T. Henriques, Christina I. Schroeder

**Affiliations:** Institute for Molecular Bioscience, the University of Queensland, Brisbane, Queensland 4072, Australia; joanna.agwa@uqconnect.edu.au (A.J.A.); y.huang@imb.uq.edu.au (Y.-H.H.); d.craik@imb.uq.edu.au (D.J.C.); s.henriques@imb.uq.edu.au (S.T.H.)

**Keywords:** Na_V_1.7, nuclear magnetic resonance, pain, rational drug design, serum stability, spider venom

## Abstract

Spider gating modifier toxins (GMTs) are potent modulators of voltage-gated ion channels and have thus attracted attention as drug leads for several pathophysiological conditions. GMTs contain three disulfide bonds organized in an inhibitory cystine knot, which putatively confers them with high stability; however, thus far, there has not been a focused study to establish the stability of GMTs in physiological conditions. We examined the resistance of five GMTs including GpTx-1, HnTx-IV, HwTx-IV, PaurTx-3 and SgTx-1, to pH, thermal and proteolytic degradation. The peptides were stable under physiological conditions, except SgTx-1, which was susceptible to proteolysis, probably due to a longer C-terminus compared to the other peptides. In non-physiological conditions, the five peptides withstood chaotropic degradation, and all but SgTx-1 remained intact after prolonged exposure to high temperature; however, the peptides were degraded in strongly alkaline solutions. GpTx-1 and PaurTx-3 were more resistant to basic hydrolysis than HnTx-IV, HwTx-IV and SgTx-1, probably because a shorter interconnecting loop 3 on GpTx-1 and PaurTx-3 may stabilize interactions between the C-terminus and the hydrophobic patch. Here, we establish that most GMTs are exceptionally stable, and propose that, in the design of GMT-based therapeutics, stability can be enhanced by optimizing the C-terminus in terms of length, and increased interactions with the hydrophobic patch.

## 1. Introduction

Gating modifier toxins (GMTs), a class of disulfide-rich peptides expressed in the venom of spiders, modulate the gating mechanism (opening and closing of an ion conduction pore), of voltage-gated ion channels [1,2,3]. Human voltage-gated ion channels are involved in several pathophysiological conditions, including chronic pain, epilepsy, and cardiovascular conditions, and, accordingly, GMTs have potential as drug leads [4,5,6,7,8,9,10,11].

**Figure 1:**
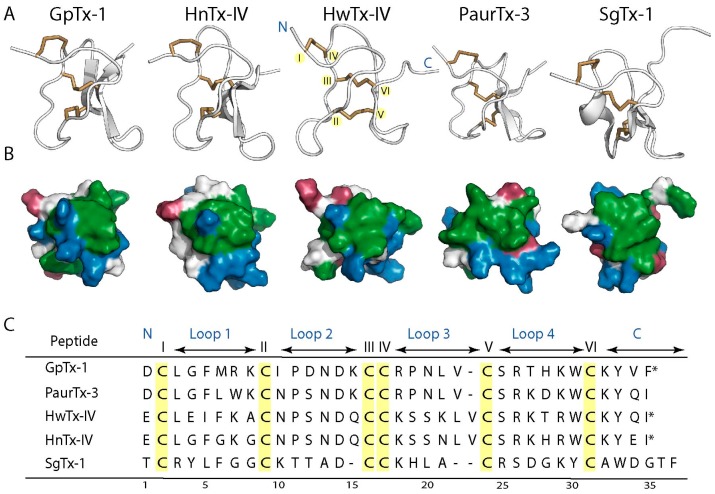
Conserved structural features of spider-derived GMTs. (**A**) ribbon representations of GpTx-1 [17], HnTx-IV (PDB ID: 1NIY) [19], HwTx-IV (PDB ID: 2M4X) [21], PaurTx-3 (PDB ID: 5WE3, this study) and SgTx-1 (PDB ID: 1LA4) [18]. The backbones of the GMTs are shown in white and the disulfide bridges forming the inhibitory cystine knot are shown in brown. GpTx-1, HnTx-IV and PaurTx-3 each have two anti-parallel β-sheets, SgTx-1 has three, and HwTx-IV has no anti-parallel β-sheets. Locations of N- and C-termini and of Cys I–VI (highlighted in yellow) are identified on HwTx-IV for clarity; (**B**) surface representations of the GMTs showing the conserved hydrophobic patch and charged ring of spider toxins, where hydrophobic residues are green, positively charged residues are blue and negatively charged residues are red. The disulfide bridges are buried within the hydrophobic patch; (**C**) sequences of the peptides are shown (aligned to cysteine residues of HwTx-IV and HnTx-IV) with cysteines highlighted in yellow, residues making up interconnecting loops are identified with arrows, N- and C-termini are shown and * denotes amidated C-terminal.

Spider GMTs are classified within the inhibitory cystine knot (ICK) family of peptides because of the presence of a conserved disulfide bridge connectivity consisting of Cys I–Cys IV, Cys II–Cys V and Cys III–Cys VI [12,13,14,15,16]. Furthermore, some ICK GMTs including GpTx-1, HnTx-IV, PaurTx-3 and SgTx-1 contain two to three antiparallel β-sheets as is expected for ICK peptides (Figure 1A) [13,14,15,16,17,18,19]. However, this is not a conserved structural feature, as some GMTs, including HwTx-IV, do not display β-sheets, exemplifying the diversity in structures of peptides containing the ICK motif (Figure 1A) [12,20,21]. In addition to the ICK motif, a second conserved feature of the structures of GMTs is a hydrophobic patch surrounded by a charged ring of amino acids (Figure 1B) [17,19,21,22,23,24]. This amphipathic characteristic, similar to that of membrane active antimicrobial peptides [25], is thought to facilitate GMT interactions with both the voltage-gated ion channels and the lipid membrane in which the channels are embedded [12,20,26,27,28,29,30,31].

It is generally assumed that GMTs have high stability; however, the ability of these ICK peptides to maintain their structural integrity in physiologically relevant conditions has not been studied in a systematic manner, though a recent study looked at the stability of Hv1a in the context of the development of insecticides [32]. The current study was designed to determine whether the ICK motif engenders GMTs with stability against thermal degradation, pH dependent hydrolysis, proteolysis and chemical degradation using GpTx-1, HnTx-IV, HwTx-IV, PaurTx-3 and SgTx-1 as model GMTs (see Figure 1C for sequences). GpTx-1, HnTx-IV and HwTx-IV are potent inhibitors of the voltage-gated sodium type 1.7 channel (Na_V_1.7), a channel that has been implicated in chronic pain and PaurTx-3 and SgTx-1 are modulators of Na_V_1.2 [33,34], a sodium channel associated with epilepsy and ataxia [1].

Here we show that GpTx-1, HnTx-IV, HwTx-IV, and PaurTx-3 are indeed stable in physiologically relevant conditions; however, SgTx-1 is susceptible to proteolysis. Our results show that, although a majority of GMTs provide excellent scaffolds for drug development, the length of the C-terminus may affect stability and needs to be considered if GMTs are to be used as templates in drug design.

## 2. Results

### 2.1. Solution NMR Structure of PaurTx-3

**Figure 2:**
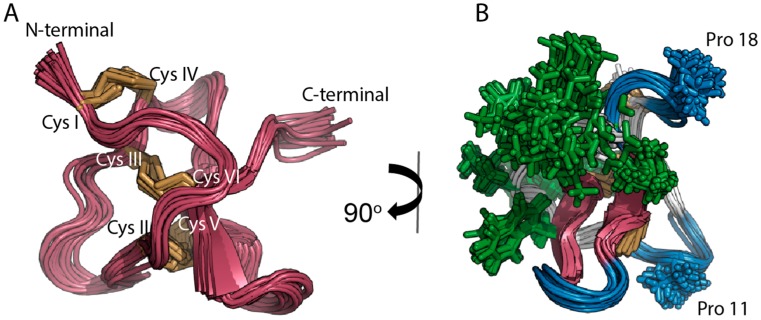
Solution NMR structure of PaurTx-3 (PDB ID: 5WE3, this study). The 20 best conformers selected from lowest energy and best MolProbity scores are shown [35]. (**A**) Backbone of the structures is red and disulfide bridges are yellow with N- and C-termini and cysteines I-VI labeled; (**B**) two antiparallel β-sheets are shown in red, the residues forming the hydrophobic patch are shown in green and the turns on the structures are shown in blue with proline residues in position 11 and 18.

Three-dimensional structures of the peptides were used to evaluate the relationship between peptide structure and stability. The structures of HnTx-IV (PDB ID: 1NIY) [19], HwTx-IV (PDB ID: 2M4X) [21] and SgTx-1 (PDB ID: 1LA4) [18] were available from the protein data bank (PDB), and PDB coordinates for GpTx-1 were made available to us as a gift [17]. The three-dimensional solution structure of PaurTx3 was not available from the PDB website and therefore calculated using nuclear magnetic resonance (NMR) spectroscopy (Figure 2) including 350 distance restraints, comprising 134 intraresidue (*i* − *j* = 0), 120 sequential (*i* − *j* = 1), 42 medium range (*i* − *j* < 5), 40 long range (*i* − *j* > 5) and 14 hydrogen bond restraints and 41 dihedral restraints including 18 ф, 18 ψ and 5 χ1 angle restraints. Root Mean Square Deviation (RMSD) values for the 20 lowest energy structures superimposed where global backbone = 0.88 ± 0.23 Å; global heavy = 1.67 ± 0.20 Å (residues 1–34) and global backbone = 0.71 ± 0.21 Å; global heavy = 1.51 ± 0.21 Å (residues 2–31, excluding flexible termini) (Table 1).

The structure comprises the expected ICK disulfide bond connectivity of Cys I–Cys IV, CysII–Cys V and Cys III–Cys VI (Figure 2A) and two antiparallel β-sheets between Val 22–Ser 24 and Lys 28–Lys 31 (Figure 2B). The structure also includes a high density of solvent exposed hydrophobic residues flanked by a series of loops and turns (Figure 2B). Two of the turns are stabilized by proline residues, whereas the third loop lacks a proline residue, probably facilitating flexibility for interactions with target voltage-gated ion channels (Figure 2B).

### 2.2. Thermal Stability

**Figure 3:**
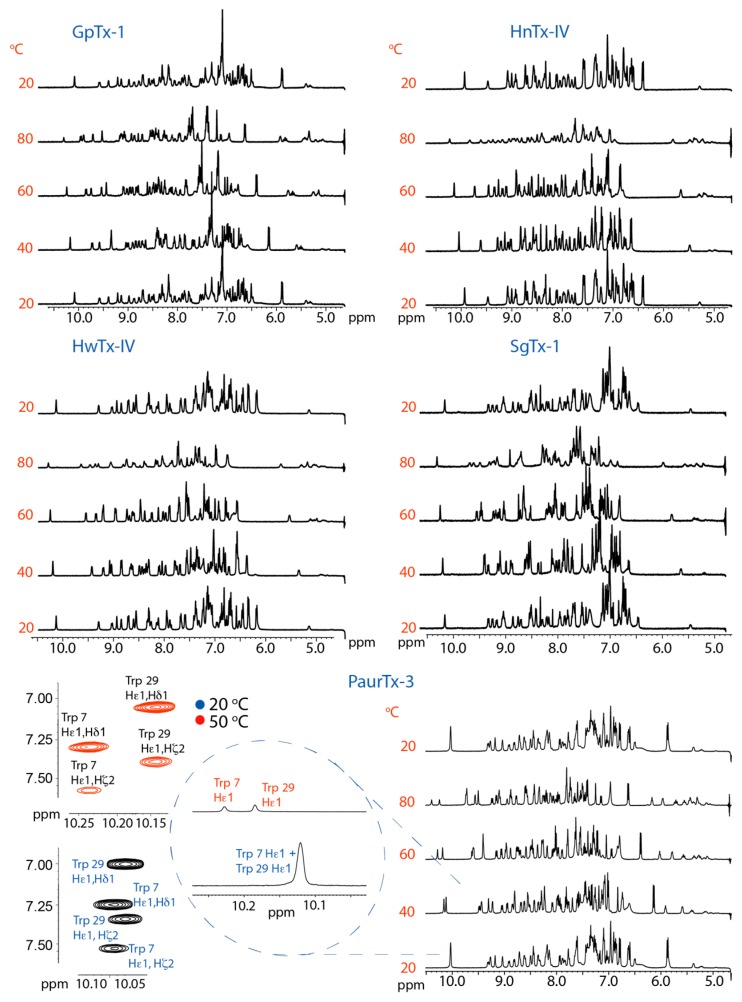
Reversible thermal denaturation of GMTs. 1D ^1^H solution NMR was used to monitor the structural changes to the five peptides at temperatures ranging from 20 to 80 °C. GpTx-1, HwTx-IV and PaurTx-3 maintained narrow peaks up to 80 °C, although there was some loss in peak intensity in the amide region of the spectra. SgTx-1 and HnTx-IV showed both a loss in intensity and broadening of peaks at 80 °C. NOE correlations confirm that the appearance of two peaks in the 10 ppm region of the 1D ^1^H spectra of PaurTx-3 are a result of the separation of the ε1 proton on the imidazole rings of Trp 29 and Trp 7.

Effects of varying temperature on peptide structure were examined using one-dimensional (1D) NMR spectra at temperatures ranging from 20 to 80 °C. The spectra (Figure 3) show peaks with narrow line widths and minimal loss in signal intensity up to 80 °C, at which temperature HnTx-IV and SgTx-1 showed peak broadening and loss of intensity. GpTx-1, HwTx-IV and PaurTx-3 maintained narrow peaks but also showed some loss in intensity at 80 °C. Changes in the chemical shifts for all the peptides were reversible, as cooling back to 20 °C resulted in spectra identical to the initial ones, at 20 °C, with narrow peaks and no loss of signal (Figure 3). Notably, for PaurTx-3, the ε1 proton shifts on the two imidazole side chains of Trp 7 and Trp 29 begin to split with increasing temperature (Figure 3 inset). Therefore, nuclear Overhauser effect (NOE) correlation peaks of PaurTx-3 were compared at 20 °C and 50 °C to confirm that the appearance of a second peak was due to a separation of Trp 7 and Trp 29 and not a result of cis-trans proline isomerization of the peptide (Figure 3 inset). In summary, the reversibility of the chemical shift changes and the well-dispersed peaks in the amide regions of the spectra of all five GMTs at each temperature level indicate that the peptides remain structured and have good thermal stability.

**Figure 4:**
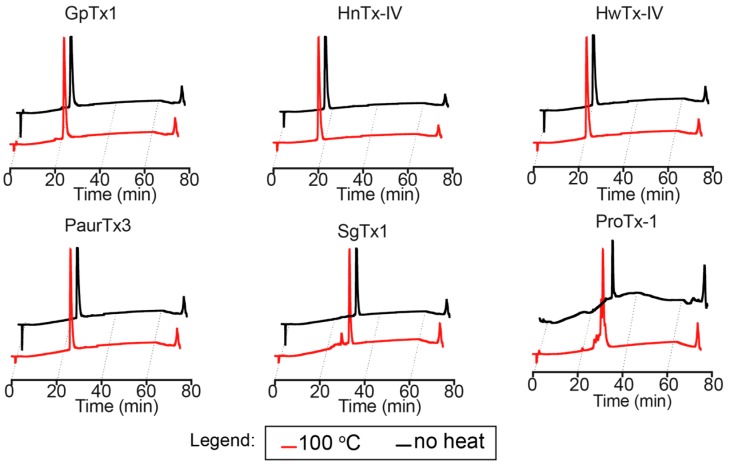
Analytical RP-HPLC traces of the GMTs following heating to 100 °C. All of the peptides except SgTx-1 and ProTx-1 showed remarkable stability after the thermal assault.

**Figure 5:**
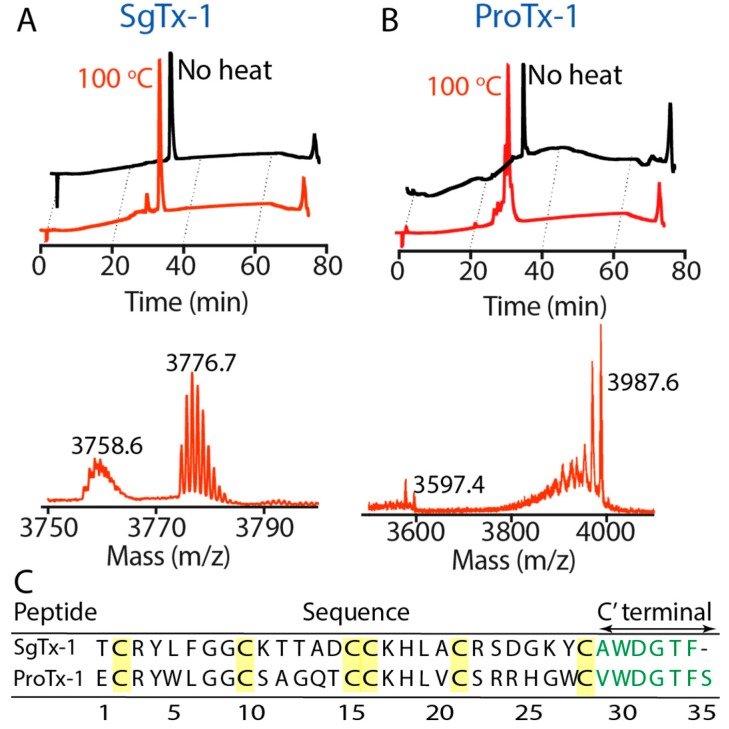
Irreversible thermal degradation of SgTx-1 and ProTx-1. Analytical RP-HPLC was used to examine the peptides after heating to 100 °C and subsequent cooling to room temperature. Degradation products were further characterized using MALDI-MS (**A**) SgTx-1 lost 18 Da from the parent peptide; (**B**) ProTx-1 underwent more extensive degradation, showing a 389.4 Da loss; (**C**) sequences of SgTx-1 and ProTx-1 are shown with cysteines highlighted in yellow and C-terminal residues shown in green.

The peptides were also subjected to more extreme temperature by boiling (100 °C) for 30 min and their stability was examined using analytical reverse-phase high performance liquid chromatography (RP-HPLC). GpTx-1, HnTx-IV, HwTx-IV and PaurTx-3 could withstand the high temperatures, as was demonstrated by identical analytical traces with and without heat treatment (Figure 4), whereas analytical traces of SgTx-1 showed a minor degradation product (Figure 5A). The degradation product from SgTx-1 had an 18 Da loss from the molecular weight of native SgTx-1 (3776.7 Da) as determined by matrix-assisted laser desorption/ionization mass spectrometry (MALDI-MS) (Figure 5A). We suspected that the longer C-terminal chain (six amino acids from the last cysteine in the sequence) of SgTx-1 made this GMT susceptible to degradation; therefore, ProTx-1, a GMT that is one amino acid residue longer than SgTx-1 was similarly subjected to 100 °C for 30 min resulting in two prominent degradation product masses, one of which was equivalent to the loss of 390.2 Da from the molecular weight of native ProTx-1 (3987.6 Da) (Figure 5C). This most likely involved the sequential hydrolytic cleavage of three ProTx-1 C-terminal residues, Thr 33, Phe 34 and Ser 35 (combined Mw = 389.4 Da) (see Figure 5C for sequence).

### 2.3. pH Dependent Hydrolysis

**Figure 6:**
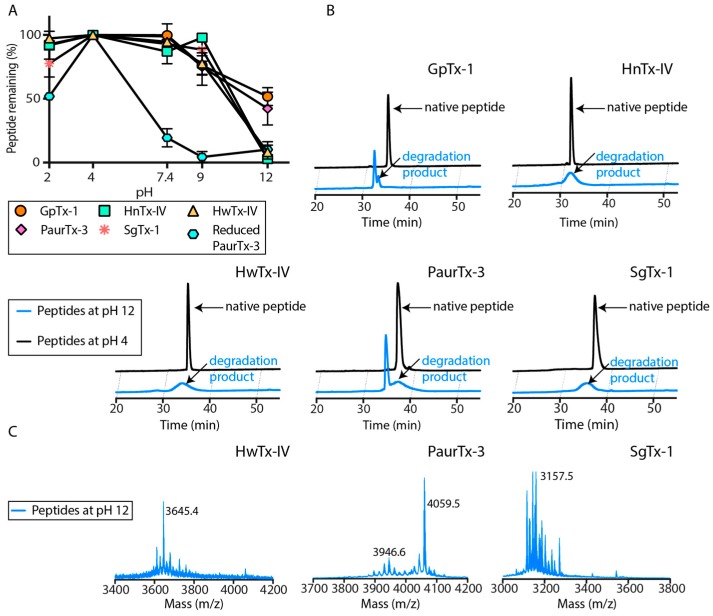
(**A**) pH dependent hydrolysis of the peptides was monitored at pH 2, 4, 7.4, 9 and 12 using analytical RP-HPLC following a 24 h incubation at 37 °C. Reduced PaurTx-3 was used as a control. Data points are relative to amount of peptide at pH 4 and error bars are ± SE for *n* = 3; (**B**) comparisons of the analytical RP-HPLC traces of the peptides at pH 4 and pH 12 are shown and (**C**) MALDI-MS spectra for HwTx-IV, PaurTx-3 and SgTx-1 at pH 12 are also shown.

All five peptides in the current study were stable when incubated at 37 °C in phosphate buffer adjusted to pH 2, 4, 7.4 and 9, with more than 75% peptide remaining after the 24 h incubation (reduced PaurTx-3 was used as a control) (Figure 6A). At pH 12, all of the peptides were partially or fully degraded, as shown in the analytical RP-HPLC traces of the peptides at pH 12 compared to pH 4 (Figure 6B). GpTx-1 and PaurTx-3 had a larger proportion of the folded peptide remaining in strong alkaline conditions compared to the HnTx-IV, HwTx-IV and SgTx-1 (Figure 6B). Analysis of the degradation products of SgTx-1, HwTx-IV and PaurTx-3 are shown as examples (Figure 6C), and reveal that the masses of the native for SgTx-1 (3776.7 Da) and HwTx-IV (4106.6 Da) were absent, whereas the mass of parent PaurTx-3 (4059.5 Da) was still observable. Analysis of the degradation products for PaurTx-3 shows a mass loss of 112.9 Da corresponding to the loss of Ile 34, and HwTx-IV shows a loss of 461 Da, within a range corresponding to the sequential hydrolytic cleavage of Ile 35, Gln 34 and Tyr 33 (458.5 Da).

### 2.4. Proteolytic Degradation

**Figure 7:**
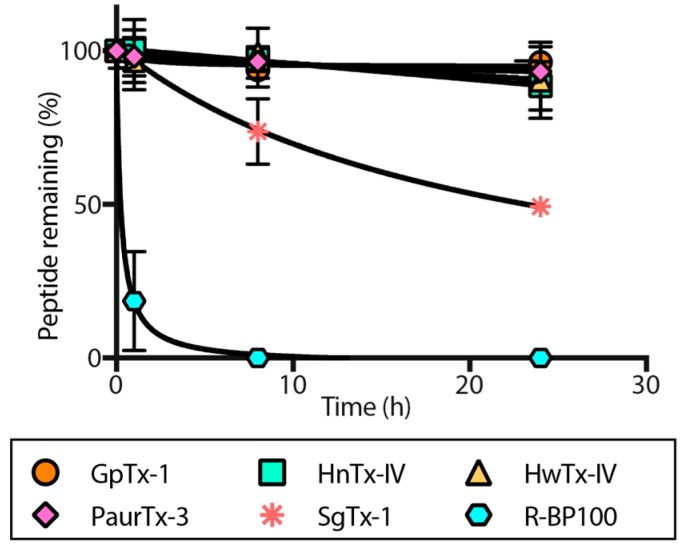
Proteolytic degradation of GMTs. The five GMTs were incubated in human serum and degradation was monitored using RP-HPLC at 0 h, 1 h, 8 h and 24 h. R-BP100 was used as a control. Data points are relative to amount of peptide at 0 h and error bars are ± SE for *n* = 3.

Stability of the peptides against proteolytic degradation in human serum was examined at 0 h, 1 h, 8 h and 24 h upon incubation at 37 °C. R-BP100 [37], a linear peptide rich in positively charged residues that does not contain disulfide bonds and is therefore likely to be susceptible to proteolyic degradation, underwent rapid proteolytic degradation to less than 20% peptide remaining after 1 h, and was completely degraded in 8 h (Figure 7). In contrast, HwTx-IV, HnTx-IV, GpTx-1 and PaurTx-3 were resistant to proteolysis with more than 90% peptide remaining after a 24 h incubation in human serum (Figure 7). SgTx-1 was degraded to approximately 70% peptide remaining in 8 h, and to approximately 50% peptide remaining in 24 h.

### 2.5. Chaotropic Degradation

**Figure 8:**
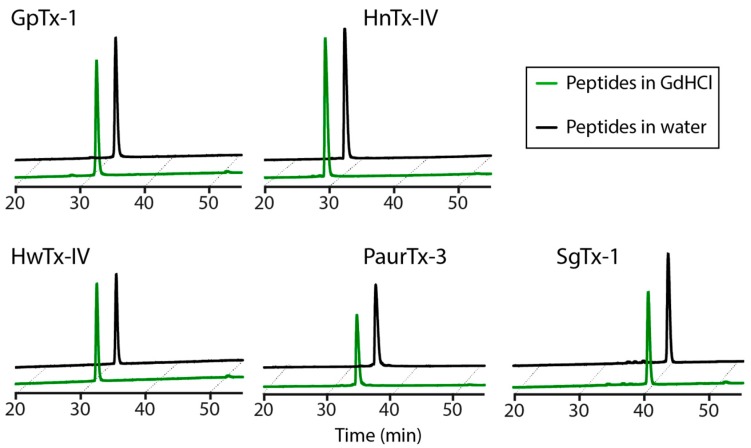
GMT stability in 6 M GdHCl following 16 h incubation at 25 °C, as monitored using RP-HPLC.

Resistance of the peptides to chemical degradation was examined by incubating the GMTs in 6 M guanidine hydrochloride (GdHCl) at 25 °C for 16 h. Each peptide displayed an identical analytical trace in the presence or absence of 6 M GdHCl upon monitoring using RP-HPLC (Figure 8).

### 2.6. Root-Mean-Square Deviation at the C-Termini GMTs

Global RMSDs of the backbones of the amino acids forming the C-terminus (comprising amino acids from the last cysteine to the C-terminal residue) of the GMTs were calculated using MOLMOL [36]. GpTx-1 and PaurTx-3 had relatively less disordered C-terminal backbones than HwTx-IV, HnTx-IV and SgTx-1 (Table 2). This low disorder is believed to reflect decreased flexibility in this region of GpTx-1 and PaurTx-3, since other factors that can contribute to disorder, such as paucity of NOEs, do not apply for these two peptides.

## 3. Discussion

This study set out to examine the stability of spider-derived GMTs under thermal, pH-dependent, proteolytic and chemical conditions. We focused on GpTx-1, HnTx-IV, HwTx-IV, PaurTx-3 and SgTx-1, which like other peptides in their class, contain the conserved ICK motif that stabilizes a solvent exposed hydrophobic patch surrounded by a charged ring of amino acid residues [15,16,17,19,21,22,23,24,38]. We were primarily interested in examining whether GMTs withstand physiologically relevant conditions; however, we also subjected the peptides to extreme thermal, pH and chemical assault to gauge the extent of the overall stability of spider-derived GMTs.

The five peptides in the current study display high thermal stability, as is illustrated by the reversible changes to their 1D ^1^H NMR chemical shifts after heating to 80 °C in the instrument and subsequent cooling back to 20 °C. Only SgTx-1 was degraded when the peptides were exposed to prolonged, extreme heating and on comparing the structure of this GMT to the remaining four peptides, we hypothesized that the longer, more flexible C-terminus of SgTx-1 (six amino acids for SgTx-1 compared to four amino acids for the remaining GMTs) may have an impact on the lower stability of the peptide. To further examine this hypothesis, ProTx-1, a GMT with one additional amino acid residue at the C-terminus compared to SgTx-1, was exposed to the same prolonged heat treatment, and we observed an even greater extent of degradation for ProTx-1 compared to SgTx-1. Furthermore, C-terminal NOEs for SgTx-1 and ProTx-1 are broad and less intense, compared to the other peptides included in this study, suggesting a more disordered C-terminal. The C-terminal residues in SgTx-1 and ProTx-1 also and have fewer long-distance interactions to hydrophobic residues in other loops compared to GpTx-1, PaurTx-3, HnTx-IV and HwTx-IV (as observed by the lack of long-range NOEs), which may explain their susceptibility to thermal degradation. Similarly, recent work on Hm3a and PcTx1, two spider ICK peptides with high sequence homology, found that Hm3a, which contains four C-terminal amino acid residues, has higher thermal stability compared to PcTx1, which has seven C-terminal amino acid residues [39]. OAIP, another spider ICK peptide containing three C-terminal amino acid residues, previously showed thermal stability [40]; however, PVIIA, an ICK toxin extracted from cone snail venom containing only one C-terminal residue, was irreversibly denatured at 56 °C [38]; therefore, it appears that a C-terminus of 3–4 amino acids may be optimal for the stability of ICK peptides against thermal assault.

**Figure 9:**
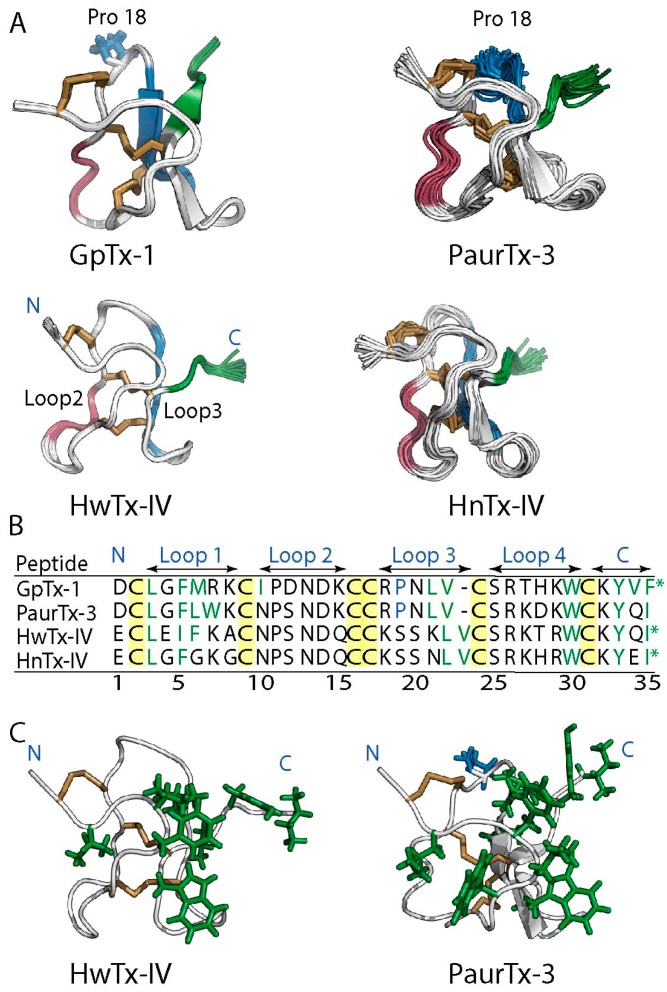
Structural features of the loops, turns and C-termini of GMTs. (**A**) ribbon representations of 20 structures of PaurTx-3 (PDB ID: 5W3E, this study), HwTx-IV (PDB ID: 2M4X) [21], and HnTx-IV (PDB ID: 1NIY) [19], and 10 structures of GpTx-1 [17], are shown where loop 2 is highlighted in red, loop 3 is in blue and the C-terminal is in green (labels on HwTx-IV for clarity). The side chain of Pro 18 is shown for GpTx-1 and PaurTx-3 in blue; (**B**) sequences of the peptides are also shown and the residues forming each loop and the C-terminal are identified by arrows. Sequences of hydrophobic amino acid residues and residues forming the hydrophobic patch are colored green and * denotes amidated C-terminal; (**C**) green sticks show the side chains of hydrophobic residues on HwTx-IV and PaurTx-3.

We examined the stability of the peptides at pH values representing various sites in the body including pH 2 (stomach), pH 4 (jejunum), pH 7.4 (plasma) and pH 9 (approximate ileum pH). pH 12 was used to represent the peptides in an extreme (non-physiological) environment. The results suggest that the peptides can withstand physiological pH ranges, with SgTx-1 perhaps being the least favorable because of the small loss of peptide at pH 2 (Figure 6A). The peptides began to show degradation at pH 9, but the most substantial degradation occurred at pH 12. Previous studies on spider-derived ICK peptides have reported that disulfide bond shuffling occurs in alkaline conditions [32,40]. However, the degradation products of the GMTs in the present study contained mass losses most probably from the C-terminus of the peptides (Figure 6B,C), and disulfide bond shuffling was not observed. Assuming that SgTx-1 was susceptible to the alkaline hydrolysis because of the length of the C-terminal on this GMT, we were curious to find out why the four remaining peptides each containing four C-terminal amino acids showed different behavior from each other when incubated at pH 12. There are three key structural features on the peptides that could withstand the alkaline assault within GpTx-1 and PaurTx-3, which are absent on HwTx-IV and HnTx-IV (which were completely degraded). First, a shorter loop 3, second, the presence of Pro 18 in the turn between loop 2 and loop 3 and, third, less disorder across the C-terminal residues (Figure 9, Table 2). It is possible that this combination of structural features facilitates a stronger attraction of the hydrophobic C-terminal residues of PaurTx-3 and GpTx-1 to the hydrophobic patch of the peptides, providing some protection from alkaline hydrolysis (Figure 9), whereas peptides like HwTx-IV and HnTx-IV, lacking this additional structural rigidity and subsequent hydrophobic attraction, become more susceptible to alkaline hydrolysis.

To consider GMTs as viable drug leads, stability of the peptides in human serum is essential. GpTx-1, PaurTx-3, HwTx-IV and HnTx-IV show exceptional serum stability (Figure 8); however, SgTx-1 is susceptible to proteolytic degradation. This degradation of SgTx-1 is most likely due to the relatively longer C-terminal, making the peptide more susceptible to proteolysis in comparison to the other GMTs in the current study.

As a final analysis of the overall stability of disulfide-rich GMTs, we examined the ability of the peptides to withstand the chaotropic effects of GdHCl. GdHCl has the potential to interfere with the integrity of peptide structures via interactions either with the backbone of the peptides or through π-cation interactions with the solvent exposed aromatic side chains of the solvent exposed hydrophobic amino acid residues [41,42]. The five GMTs in the current study were unaffected by the chemical assault, most likely because of the presence of the ICK motif. A previous study on AS-48, a globular cyclic peptide also containing a hydrophobic surface patch, but lacking disulfide bridges, showed that the peptide was degraded by 6.3 M GdHCl at 25 °C [43]. Cyclic peptides containing the ICK motif were subsequently found to be stable when subjected to GdHCl [38]. Therefore, we conclude that disulfide bridges confer chemical stability to spider-derived ICK GMTs.

## 4. Conclusions

In conclusion, GpTx-1, HwTx-IV, HnTx-IV and PaurTx-3 are stable in physiologically relevant environments. SgTx-1 underwent partial degradation when subjected to proteolytic, acidic (pH 2) and extreme temperatures, probably because of the length of the C-terminal of this particular peptide. To avoid similar degradation in the use of GMTs as templates for drug design, a three to four residue C-terminal appears to be preferable for stability. Hydrophobic interactions between the C-terminal chain and the hydrophobic patch on the surface of the peptide may provide additional stability to GMTs against hydrolysis in strongly alkaline solutions, and these hydrophobic interactions can be augmented by designing GMT analogues with a shorter loop 3 stabilized by a proline on the turn between loop 2 and 3.

Spider-derived GMTs are undeniably among nature’s more interesting pharmacological probes in the study of voltage-gated ion channels [6,12,34]. The current work has provided additional insight into the potential of these ICK peptides as templates for drugs designed to target ailments linked to the voltage-gated ion channels.

## 5. Materials and Methods

### 5.1. Peptide Synthesis

Peptides were synthetically assembled using automated solid phase peptide synthesis on a Symphony peptide synthesizer (Protein Technologies Inc., Tucson, AZ, USA), as previously described [26]. Side chain protecting groups were removed and the reduced peptides were released from the resin using 96% (*v*/*v*) trifluoroacetic acid (TFA), 2% (*v*/*v*) water and 2% (*v*/*v*) triisopropylsaline (TIPS) as before [26].

Oxidative folding of PaurTx-3 and GpTx-1 was achieved in 16 h at room temperature in 7.5% (*v*/*v*) acetonitrile (ACN), 0.1 M Tris(hydroxymethyl)aminomethane, 0.81 mM reduced glutathione (GSH) and 0.81 mM oxidized glutathione (GSSG) at pH 7.7 [17]. ProTx-1 and SgTx-1 were oxidized over 72 h at 4 °C in a buffer containing 0.1 M ammonium acetate, 2 M urea, 2.5 mM GSH and 0.25 mM GSSG at pH 7.8 [18]. HnTx-IV and HwTx-IV were oxidized at room temperature for 16 h in a buffer containing 0.1 M Tris, 5 mM GSH, 0.5 mM GSSG ph 8, oxidation of HnTx-IV additionally required 0.1 M NaCl in the buffer solution [26,44,45]. The peptides were purified using RP-HPLC on a C18 column using preparatory (8 mL/min) and semi-preparatory (3 mL/min) flow rates on gradients obtained using solvent A (0.05% TFA in water) and solvent B (0.05% TFA in 90% ACN) as previously described [26].

### 5.2. NMR Structure Calculation for PaurTx-3

Data used for PaurTx-3 structure calculations was acquired on a Bruker Avance 600 MHz NMR spectrometer (Bruker, Billerica, MA, USA) equipped with a cryoprobe. The solution NMR structure for PaurTx-3 was calculated using previously described protocols [26], on a Bruker Avance 600 MHz NMR spectrometer equipped with a cryoprobe. CCPNMR Analysis 2.4.1 (CCPN, University of Cambridge, Cambridge, UK) was used for amino acid assignment [46,47]. The solution NMR structure for PaurTx-3 was calculated as previously described [26] using the AUTO and ANNEAL functions in CYANA 3.97 (Güntert Group, Goethe-Univerity Frankfurt, Frankfurt, Germany) [48] to refine peak assignments. Dihedral angle restraints were generated using TALOS-N (Bax Group, NIH, Pike Bethseda, MD, USA) [49]. After initial structure determination on CYANA, protocols on the RECOORD database [50], were used to generate 50 structures which were then refined in a water shell [51], and a final set of 20 structures was chosen based on the lowest energy, best MolProbity scores [35], and fewest distance and dihedral angle violations (Table 1). PaurTx-3 structure and restraints have been submitted to the Protein data bank (PDB ID: 5WE3) and the Biomagnetic Resonance Data bank (BMRB: 30317).

### 5.3. Peptide Quantification

Stock concentrations of peptides were quantified using Nanodrop at 280 nm using extinction coefficient (ε_280_) values as follows: GpTx-1 ε_280_ = 7365 M^−1^ cm^−1^; HnTx-IV ε_280_ = 7365 M^−1^ cm^−1^; HwTx-IV ε_280_ = 7365 M^−1^ cm^−1^; PaurTx-3 ε_280_ = 12,865 M^−1^ cm^−1^; SgTx-1 ε_280_ = 8855 M^−1^ cm^−1^. Unless otherwise stated, stock concentrations of the peptides were 300 μM in water.

### 5.4. Analytical RP-HPLC

For experiments monitored using RP-HPLC, solvent A (0.05% TFA in water) and solvent B (0.05% TFA in 90% ACN) were used on a C18 phenomenex column at a flow rate of 0.3 mL/min using a 1% gradient of 0–50% solvent B and monitored at 215 nm.

### 5.5. Thermal Stability

A Bruker 500 MHz Avance nuclear magnetic resonance (NMR) spectrometer was used to heat the peptides (1 mg/mL in 9:1 *v*/*v* H_2_O/D_2_O) and to monitor structural changes at temperatures ranging from 20 to 80 °C. One-dimensional (1D) ^1^H NMR spectra and two-dimensional (2D) nuclear Overhauser effect spectroscopy (NOESY) (200 ms mixing time) spectra were acquired and processed using TopSpin 3.5 (Bruker, Billerica, MA, USA) and CCPNMR Analysis 2.4.1 was used in the assignment of the NOE spectra [46,47].

Peptides were also subjected to boiling (100 °C) in water for 30 min using a heating block, cooled to 25 °C and analyzed using analytical RP-HPLC. The mass of the degradation products from SgTx-1 and ProTx-1 were determined using matrix-assisted laser desorption/ionization mass spectrometry (MALDI-MS).

### 5.6. pH Dependent Hydrolysis

The ability of the peptides to withstand varying pH conditions was monitored by incubating the peptides (30 μM) at 37 °C in phosphate buffer adjusted to pH 2, 4, 7.4, 9 and 12 using phosphoric acid and/or sodium hydroxide. After 24 h, each sample was adjusted to pH 2 using phosphoric acid and analytical RP-HPLC was used to quantify the percentage of peptide remaining by examining the height of the peaks at each pH relative to pH 4 (the pH where most samples showed highest stability). Reduced PaurTx-3 was used as a control.

### 5.7. Proteolytic Degradation

Human serum isolated from male AB plasma was used to examine the stability of the peptides to proteolysis. Peptides (30 μM) were incubated with human serum or phosphate buffered saline (PBS) at 37 °C and reactions were stopped at 0, 1, 8 or 24 h by placing the samples on ice, 6 M urea was used to denature the proteases and 20% trichloroacetic acid was used to precipitate the proteases. Peptides were separated from the proteases by centrifugation at 17,000 *g* for 10 min. Effects of proteolysis were examined using RP-HPLC, whereby retention times of the peptides were determined using the PBS controls at 0 h and percentage of peptide remaining in human serum was obtained from the heights of the peaks at each time point relative to 0 h. RBP-100 was the control.

### 5.8. Chaotropic Stability

Chemical stability of the peptides (12 μM) was studied by incubating them in 6 M GdHCl at 25 °C for 16 h. Analytical RP-HPLC was used to compare the peptides in GdHCl to peptides in water. RBP-100 was used as a control.

### 5.9. RMSD Calculation

MOLMOL (Version 2k.2, Institute of Molecular Biology and Biophysics, ETH, Zürich, Switzerland) [36] was used to calculate the global atomic RMSDs of the backbone of the GMTs including the amino acid residue following the last cysteine to the C-terminal of each sequence. RMSD values of the amino acids prior to the final cysteine were also calculated.

## Figures and Tables

**Table 1:** Energies and structural statistics ^1^ for the final 20 ^2^ structures of PaurTx-3.

**Energies (kcal/mol)**	
Overall	−1232.31 ± 50.68
Bonds	23.83 ± 2.03
Angles	64.83 ± 6.52
Improper	19.89 ± 2.47
Dihedral	164.27 ± 1.66
Van der Waals	−131.51 ± 7.15
Electrostatic	−1374.68 ± 52.43
NOE	0.38 ± 0.04
Constrained dihedral (cDih)	0.66 ± 0.40
**MolProbity Statistics**	
Clash score (>0.4 Å/1000 atoms)	11.10 ± 3.94
Poor rotamers (%)	2.88 ± 2.30
Ramachandran outliers (%)	0.47 ± 1.14
Ramachandran favoured (%)	87.50 ± 3.36
MolProbity score	2.45 ± 0.26
MolProbity percentile ^3^	50.40 ± 14.91
**Atomic RMSD (Å)**	
Mean global backbone (2–31) ^4^	0.71 ± 0.21
Mean global heavy (2–31)	1.51 ± 0.21
Mean global backbone (1–34)	0.88 ± 0.23
Mean global heavy (1–34)	1.67 ± 0.20
**Distance Restraints**	
Intraresidue (*i* − *j* = 0)	134
Sequential (*|i* − *j|* = 1)	120
Medium range (*|i* − *j|* < 5)	42
Long range (*|i* − *j|* > 5)	40
Hydrogen bonds ^5^	14
Total	350
**Dihedral Angle Restraints**	
φ	18
ψ	18
χ1	5
Total	41
**Violations from Experimental Restraints**	
Total NOE violations exceeding 0.2 Å	1
Total dihedral violations exceeding 2.0°	2

^1^ ±St Dev. ^2^ Based on structures with highest overall MolProbity score [35]. ^3^ 100th percentile is the best among structures of comparable resolution; 0th percentile is the worst. ^4^ RMSD calculated in MOLMOL (Version 2k.2, Institute of Molecular Biology and Biophysics, ETH, Zürich, Switzerland) [36]. ^5^ Two restraints were used per hydrogen bond.

**Table 2:** GMT C-terminal RMSD ^1^ values.

Peptide	Backbone RMSD (Å) (Pre C-Term) ^2^	Backbone RMSD (Å) (C-Term) ^3^
GpTx-1	0.18 ± 0.09 (Asp 1–Trp 29)	0.17 ± 0.09 (Lys 31–Phe 34)
HnTx-IV	0.60 ± 0.13 (Glu 1–Trp 30)	0.51 ± 0.22 (Lys 32–Ile 35)
HwTx-IV	0.34 ± 0.09 (Glu 1–Trp 30)	0.39 ± 0.17 (Lys 32–Ile 35)
PaurTx-3	0.79 ± 0.22 (Asp 1–Trp 29)	0.30 ± 0.14 (Lys 31–Ile 34)
SgTx-1	0.38 ± 0.11 (Thr 1–Tyr 27)	1.58 ± 0.43 (Asp 29–Phe 34)

^1^ Global RMSD calculated using MOLMOL [36]. ^2^ Residues prior to the final cysteine in the sequence ± standard deviation (SD) from the mean. ^3^ Residues after the final cysteine in the sequence ± SD from the mean.
